# Arsenic in Rice and Rice-Based Products with Regard to Consumer Health

**DOI:** 10.3390/foods13193153

**Published:** 2024-10-02

**Authors:** Monika Rajkowska-Myśliwiec, Artur Ciemniak, Gabriela Karp

**Affiliations:** Department of Toxicology, Dairy Technology and Food Storage, Faculty of Food Science and Fisheries, West Pomeranian University of Technology in Szczecin, 71-459 Szczecin, Poland; artur.ciemniak@zut.edu.pl (A.C.); biuro@dermicos.pl (G.K.)

**Keywords:** arsenic, rice and rice products, risk assessment, THQ, MoE, LCR

## Abstract

Most articles on the exposure to arsenic (As) associated with rice and rice products come from Asia where these products are consumed in the largest quantities; relatively few of the articles have focused on European consumers. Since rice products can represent a significant contribution to overall arsenic exposure, the aim of the study was to determine the total arsenic content (tAs) in rice and the most commonly-consumed rice products available on the Polish market. The tAs determination was performed by hydride generation coupled to inductively-coupled plasma optical emission spectrometry (HG-ICP-OES). Because an inorganic form of As (iAs) is mutagenic and carcinogenic and about 100-fold more toxic than the organic form, an additional aim of the study was to assess the risk of its ingestion, assuming that it constitutes 67.7%, 72.7%, or 90% of tAs. In all products tested, the calculated iAs content was below the maximum permissible levels, and no threat was found for any of the analyzed Polish consumers, based on the mean rice consumption in Poland and the mean calculated iAs content. However, a potential health risk was noted among infants and young children, assuming maximum iAs levels and threefold higher consumption (16.2 g d^−1^). To avoid a risk of developing cancer, infants up to one year of age should consume no more than 32.2 g of the studied products per week, children under three years of age up to 68.7 g, and adults 243 g. Consumers should strive to include a variety of cereals in their daily diet and choose products shown to have low arsenic contamination levels based on testing and inspection rankings.

## 1. Introduction

Rice is the seed of the monocot plants *Oryza sativa* (Asian rice) or *Oryza glaberrima* (African rice). In crop year 2022, around 165 million hectares were reserved for the cultivation of rice worldwide, with the leading global producer being India, who harvested about 48 million hectares of rice. Rice occupies 11% of the global cultivated area (Asia 24%) [1,2]. As a cereal grain, it is the most important staple food for a large portion of the global population. It is the most popular starchy food in the world, providing 2/3 of the caloric intake of over three billion people in Asia and 1/3 of the caloric intake of almost 1.5 billion people in Africa and Latin America [3]. Moreover, rice is considered one of the most economically important crops in many developing countries [3]. In the 2022/23 crop year, about 520.4 million metric tons of rice were consumed worldwide. As the most populous country in the world, China consumes more rice than any other country, about 150 million metric tons in 2023/24. Following China, India is ranked second with 118 million metric tons of rice consumed in the same period [4]. Globally, the annual mean rice consumption per person stands at 67.5 kg as of 2023. This translates into a daily rice consumption per capita of 184.9 g [5]. In developing countries, rice consumption is around 0.40–0.65 kg per person per week [6]. Asia accounts for 90% of global rice consumption, with the highest consumption by country being Bangladesh (21.4 kg/month), Laos (20.1 kg/month), Cambodia (20.8 kg/month), and Vietnam (17 kg/month) [7]. By comparison, the mean monthly consumption per person in households in Poland in 2022 was 0.15 kg, with the highest consumption among retirees and pensioners (0.17 kg) and the lowest among farmers (0.11 kg) [8].

The popularity of rice has been attributed to its bland taste, good nutritional value as a source of carbohydrates, and the absence of gluten [9]. Rice is increasingly used as an ingredient in baby foods, and toddlers consume as much as threefold more than adults in relation to their body weight [10,11]. Rice-based products are particularly attractive options during weaning and for feeding young children due to their availability, bland taste, nutritional value, and relatively low allergic potential. As such, risk assessments have reported considerable differences between adults and toddlers regarding inorganic arsenic (iAs) consumption [12].

Food constitutes a primary source of exposure to iAs for the general population in Europe. Among the various foods, the main contributors to total As and iAs were found to be grains and grain-based products, particularly rice, as indicated by the EFSA and also by the first Total Diet (TSD) study in Germany [11,13,14]. These results are consistent with the results of the report of the German Federal Institute for Risk Assessment (BfR) [15]. Furthermore, according to the above report, rice and rice products such as rice waffles and rice cream for infants may contain relatively high levels of inorganic arsenic, which is of particular toxicological concern. For example, consumers of rice cakes (one piece per day) ingest four times as much inorganic arsenic through this rice product alone, compared with the total intake of iAs in all respondents. While drinking water also contributes to the exposure, its levels are usually low in Europe [13]. Studies have found children, especially toddlers, aged 1–3 years, to be at particular risk of dietary arsenic exposure [9]; they are believed to consume two to three times as much arsenic as adults relative to their body weight as they have a comparatively greater intake of food and fluids [16]. Recent health risk assessments indicate that the consumption of arsenic-containing rice and rice-based foods (e.g., cereals, cakes, and crackers) has led to increased cancer risks, especially among infants and children [17]. Maximum levels of inorganic arsenic in rice and rice products are set in the EU Regulation No. 2023/915 [18] and are in the range of 0.10–0.30 mg kg^−1^. Numerous investigations have shown that more than 90% of the arsenic in rice grains in areas endemic for arsenic is in the inorganic form [19]. In soil, arsenic mostly exists in the form of As(V) or As(III). In flooded paddy fields, As(V) is easily reduced to As(III), which is more mobile [20]. Rice has a high ability to take up and translocate As(III) under the anoxic environment of flooded paddy soils [19]. Moreover, the rice plant has a higher ability to reduce As(V) in the root and then efficiently transport As(III) to xylem sap, resulting in high levels of As in the grain [19,21,22].

Due to it being a Group I carcinogen associated with skin, bladder, liver, kidney, and lung cancer in humans, arsenic is classified as a chemical of major public health concern [23]. As such, geogenic arsenic is a significant public health challenge and one that affects 140 million people in 70 countries around the world [24]. Arsenic is naturally present in large amounts in soil in some parts of the world, especially in Bangladesh, Argentina, Vietnam, and Thailand [15], and its effects are particularly visible in rice crops where water is the main carrier of arsenic compounds [15,25].

A recent risk assessment by the EFSA found consumer exposure to inorganic arsenic in food to be a matter of possible concern. Epidemiological data indicate that chronic intake of iAs via diet and/or drinking water is associated with an increased risk of several adverse outcomes, including skin, bladder, and lung cancer [13]. These findings confirm those of a previous EFSA assessment, in 2009, of the risks linked to the presence of inorganic arsenic in food. A risk assessment of the combined exposure to inorganic and organic arsenic will be available by 2025. Regulations on the maximum permissible levels of iAs and the recommendations for population exposure levels (BMDLs) have been introduced relatively recently [13,18]. Consequently, there is a need to update data on the exposure of consumers, especially children, to iAs and to identify rice products with low inorganic arsenic content worldwide. Moreover, the mechanism of carcinogenicity of inorganic arsenic has not yet been fully elucidated. Therefore, it is difficult to establish a safe intake amount that would not be associated with an increased risk of cancer. The presence of inorganic arsenic in food is therefore undesirable in any amount.

Taking into account the potential exposure of consumers, especially children, to dangerous levels of arsenic, the aim of this study was to determine total arsenic (tAs) and inorganic arsenic (iAs) levels in rice and selected rice-based products. It also assesses the level of inorganic arsenic intake in adults and children aged one to three years through typical consumption of rice and rice products. In addition, these studies aim to establish the maximum portion of the tested products (g week^−1^) in order to avoid the risk of cancer (LCR > 1) associated with iAs consumption. The consumption data on rice products should be updated so that exposure can be estimated realistically, especially where small children are concerned.

## 2. Materials and Methods

### 2.1. Materials

The research material consisted of the following groups of products, white rice (R), brown rice (Rb), rice cakes (C), and rice noodles (N), purchased at randomly-selected stores in Szczecin, Poland, in 2023. Most of the selected products are widely available in Polish supermarkets. A total of 120 products were purchased, i.e., five product packages from each of six manufacturers (Table 1). Each of the five products differed in batch number and/or expiration date. Sampling was carried out in accordance with Regulation (EU) 2021/705 [26]. In most cases, white and brown rice were packed in sets of four bags of 100 g in cardboard boxes. The variety of rice was not specified on the packaging. Rice cakes and rice noodles were natural products, without additives, packed in polyethylene packages, weighing 100–130 g and 200 g, respectively. Not every product contained information about the country of origin of the rice. In the case of both white and brown rice, the same producer (KP) did not provide the origin; in the case of rice cakes, such information was missing from the product labels provided by five of the six producers.

### 2.2. Methods

The analytical methods used in these studies meet the criteria contained in Commission Implementing Regulation (EU) 2021/705 [26].

#### 2.2.1. Sample Preparation

The analytical process used Merck reagents of the highest purity (Merck KGaA, Darmstadt, Germany) and deionized water from the Barnstead EASY pure UV apparatus. The initial processing of the tested material consisted of homogenization using an agate mortar. Approximately 5 g of homogeneous material was prepared from each purchased product. The homogenized material was weighed into Teflon vessels of a CEM MDS-2000 microwave digester oven (SpectraLab Scientific Inc., Markham, ON, Canada) in amounts of 0.250 ± 0.001 g, and then 6 mL of EMSURE^®^, 65% nitric acid was added. In addition, two blank samples and one reference sample (0.150 g) were also prepared for each series of 12 samples. The operating parameters of the mineralizer are listed in Table 2. After mineralization, the samples were filtered and quantitatively transferred with deionized water (0.05 μS cm^−1^; Barnstead™ Gen-Pure™ Pro, Thermo Scientific, Erlangen Germany) to polyethylene bottles, obtaining a total sample weight of 25.000 ± 0.150 g.

Laboratory quality assurance and quality control (QA/QC) included procedural blanks (reagent samples; 6 mL of 65% HNO_3_) and an analysis of Certified Reference Material, SRM 1568a, Rice Flour (National Institute of Standards and Technology (NIST), USA). The obtained results (0.30 ± 0.02 µg g^−1^; RSD = 6.7%) demonstrated close compliance with the reference value (0.29 ± 0.03 µg g^−1^). The Horrat value was 0.35, which indicates adequate precision and satisfactory repeatability [26]. Recovery was 103%. The limit of detection (LOD) was 0.3 ug kg^−1^, calculated based on three times the standard deviation (LOD = 3 × SD) of As results from 10 independent measurements of blank samples.

#### 2.2.2. Instrumental Analysis

The arsenic content was determined in previously prepared samples using the hydride generation method. The measurement was carried out in a JY-24 sequential ICP spectrometer (ICP-AES Jobin Yvon, France), operating under the conditions shown in Table 3. The hydride generation system (Hg-ICP-AES) consisted of a hydride generation unit and a Meinhard nebulizer for sample introduction to the cyclone spray chamber. A quartz plasma torch with an alumina injection tube was used. Two dual-channel peristaltic pumps were used to simultaneously introduce the solutions of the tested samples and reagents, viz. NaBH_4_ and HCl, into the hydride generator system. The gaseous sample containing As as volatile hydrides was then transported to the plasma torch. A post-reaction solution was drained from the spray chamber to waste by the peristaltic pump.

#### 2.2.3. Statistical Analysis

Statistical analysis were performed using the Statistica software package, version 13.3 (StatSoft, Krakow, Poland). The normality of the tAs distribution in all tested product groups was confirmed by the Shapiro–Wilk test. The significance of differences between the mean arsenic contents in the tested material were assessed using analysis of variance (ANOVA) and the non-parametric Duncan test and verified at the significance level of *p* ≤ 0.05.

#### 2.2.4. Arsenic Intake and Consumer Risk Assessment

The risk to human health associated with the intake of inorganic arsenic from rice and rice products was determined with regard to three groups of consumers: adults (70 kg bw, i.e., body weight) and children aged between six and 12 months (9.25 kg bw) and at three years old (15 kg bw) [27]. All standards regarding the assessment of arsenic levels in food and consumer safety refer to the inorganic, most toxic form of this element (iAs); this form has been found to represent about 70% of total arsenic (tAs) in rice but rarely exceed 85% [28]. Similar iAs levels (75.2–90.1%) have been noted previously in rice products [29], although the literature indicates the share of total As to vary considerably (22.4–97.7%) with a mean value of 72.7% (Table A1, Appendix A). Elsewhere, the mean proportion of iAs is 67.7% of tAs [25], while FDA and other studies indicate that the share of iAs is up to 90% of tAs [20,30].

These data were used to estimate the iAs content in rice and rice products. Our data were used to assess the arsenic level in the analyzed products in relation to the applicable EU regulations [18] and to conduct a consumer risk assessment based on EFSA guidelines [13]. 

Rice consumption was estimated as per capita daily intake of uncooked rice from all sources, i.e., rice grain and rice products [30]. The results of estimated daily intake (EDI, µg kg^−1^ d^−1^), target hazard quotient (THQ), margin of exposure (MoE), and lifetime cancer risks (LCR) were obtained according to the Equations (1)–(5) [31,32].

(1) $$EDI=\frac{C\times IR}{BW}$$

where C—arsenic content in rice/rice products (μg g^−1^); and IR—daily ingestion rate (g d^−1^) and BW—body weight (kg).

The estimated daily intake (EDI) values were compared to the BMDL_05_ (0.06 μg kg^−1^ bw per day) (Benchmark Dose Lower Confidence Limit) [13]. The CONTAM Panel concluded that BMDL_05_ should be considered as a Reference Point (RP) to estimate the risk of skin, lung, and bladder cancer, skin lesions, ischemic heart disease, chronic kidney disease, respiratory disease, spontaneous abortion, stillbirth, infant mortality, and neurodevelopmental effects. For comparative purposes, obtained EDI values were also related to the previously applicable BMDL_01_ value of 0.3 μg kg^−1^ bw d^−1^ [11].

The target hazard quotient (THQ) (Equation (2)) was calculated based on EDI (µg kg^−1^ d^−1^) and the reference oral dose (RfD) for iAs of 0.3 µg kg^−1^ d^−1^ [33].

(2) $$THQ=\frac{EDI}{RfD}$$

THQ < 1 signifies non-obvious risk. Conversely, an exposed population of concern will experience health risks if the dose is equal to or greater than the RfD.

The hazard index (HI) used to describe the cumulative non-carcinogenic effect (Equation (3)) in this study corresponded to the THQ because it refers to only one element.

(3) $$HI=\sum_{i=1}^{n}THQ$$

The margin of exposure (MoE) was calculated between the BMDL_05_ value for the critical tumor site(s) and the estimated consumers’ exposure levels (Equation (4)).

(4) $$MoE=\frac{{BMDL}_{05}}{EDI}$$

For comparative purposes, MoE calculations were also performed with reference to the previously-applicable BMDL_01_ value of 0.3 μg kg^−1^ bw d^−1^ [11]. An MoE of 1 describes the exposure level that could be associated with a 5% increase relative to the background incidence of skin cancer based on the available data. The MoE does not precisely quantify the health risk but gives an indication of the level of deserved concern: a smaller MoE indicates a higher potential risk posed by exposure to iAs [34].

Moreover, EDI (µg kg^−1^ d^−1^) and a slope factor (SF = 1.5 mg kg^−1^ d^−1^) were used to calculate the lifetime cancer risk (LCR) (5) [31]. The range of acceptable risk of exposure to carcinogenic substances over a lifetime is between 10^−6^ (1 in 1,000,000) and 10^−4^ (1 in 10,000).

(5) $$LCR=EDI\times SF\times 10^{-3}$$

All calculations were considered in four scenarios for assessing the risk to human health related to the intake of inorganic arsenic from rice and rice products (Table 4). The first two scenarios assumed a consumption rate of 5.4 g d^−1^, together with the mean (scenario 1) and maximum calculated iAs contents (scenario 2). Scenarios 3 and 4 assumed three times higher consumption (16.2 g kg^−1^), together with average (scenario 3) and maximum content of iAs (scenario 4). To distinguish the obtained results, the corresponding scenario numbers were added to the individual variables, e.g., in scenario 1: EDI 1; THQ 1; MoE 1, LCR 1.

## 3. Results

### 3.1. Arsenic Content in Rice and Rice-Based Products

The total arsenic (tAs) contents of the rice and rice-based products are shown in Table 5. Depending on the product, tAs ranged from 69.5 to 227.8 µg kg^−1^. The mean level of tAs varied by product and was as follows: brown rice > rice noodles > rice cakes > white rice. However, the difference between the tAs content in white rice and rice cakes was not statistically significant (Table 5). Table 5 also gives the inorganic arsenic (iAs) levels, given as a percentage share of total As content. The following values were assumed based on previous research: 67.7% [25], 72.7% (Table A1, Appendix A), or 90% [20,30].

**Figure 1:**
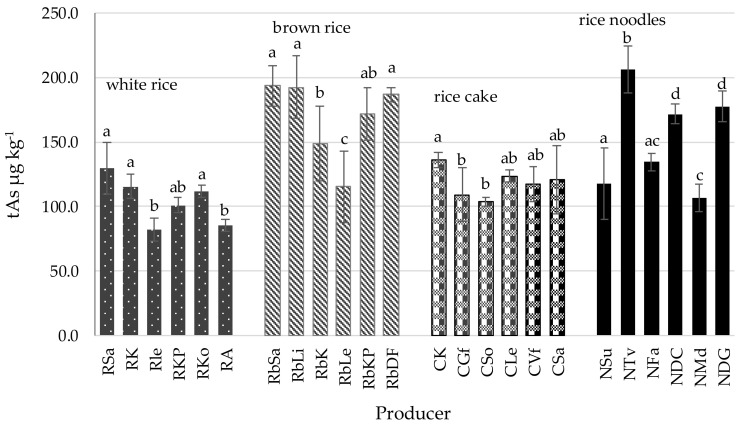
Total arsenic in rice and rice products depending on producer (Mean ± SD). Legend: R—white rice; Rb—brown rice; C—rice wafers; N—rice noodles, subsequent symbols refer to the producers; a–d Values marked with different letters indicate significant differences (Duncan test, *p* < 0.05).

Within the product groups, differences in arsenic content were found depending on the producer (Figure 1). The least significant differences (*p* < 0.05) between producers were found in the case of rice cakes, and the most significant differences were found in rice noodles.

### 3.2. Consumer Risk Assessment

The three consumer groups (adult 70 kg bw, child 15 kg bw, child 9.25 kg bw) were subjected to four risk assessment scenarios (Table 4). Scenarios 1 and 3 were based on the calculated mean iAs levels as proportions of tAs (i.e., 67.7%, 72.7%, and 90%) while scenarios 2 and 4 assumed the maximum determined level of total arsenic (tAs) in the products (Table 4). In addition, scenarios 1 and 2 assumed the average daily consumption of rice in Poland (5.4 g) [8], while scenarios 3 and 4 assumed three times greater consumption (16.2 g) (Table 4).

The results of scenario 1 are given in Table 6. Calculations for the remaining scenarios (2–4) are presented in Appendix A (Table A2, Table A3 and Table A4, Appendix A). All tables contain EDI and parameters used to assess non-carcinogenic (THQ, MoE) and carcinogenic risk (LCR) associated with inorganic arsenic intake. The mean estimated daily intake of iAs from the individual tested product groups, based on scenario 1 (EDI 1), is presented in Table 7. Mean EDI 1 values ranged from 0.008 to 0.071 µg kg^−1^ bw d^−1^, with the highest value (0.120 µg kg^−1^ bw d^−1^) recorded in the younger children (9.25 kg bw) at an iAs of 90% of total As. No statistically significant differences in iAs intake were found between consumer groups for either the 67.7% or 72.7% iAs content. However, calculations based on 90% iAs showed a significant increase in EDI in each consumer group (*p* < 0.05) (Table 6).

**Figure 2:**
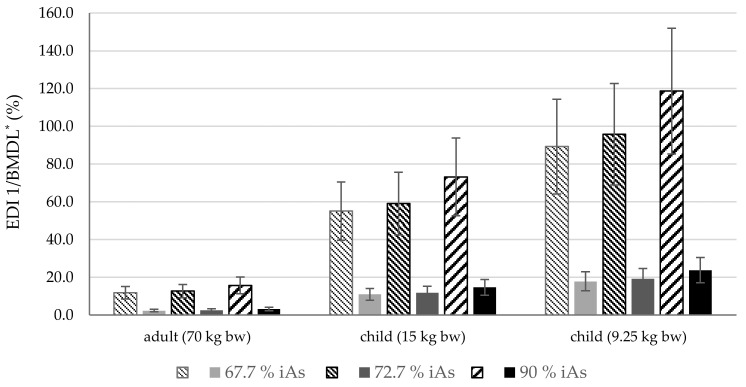
Comparison of EDI 1 results with current and previous BMDL values for iAs (Mean ± SD). * The striped bars represent the percentage share of EDI 1 in relation to the applicable BMDL_01_ [13], and the solid bars correspond to the calculations based on the previously-applicable BMDL_05_ [11]. The iAs share (inorganic arsenic) is assumed to be 67.7%, 72.7%, and 90%.

The ratio of the obtained EDI 1 values to the currently-applicable BMDL_05_ level (0.06 µg kg^−1^ d^−1^) and the previously-applicable BMDL_01_ (0.3 µg kg^−1^ d^−1^) is presented with regard to age groups in Figure 2. It can be seen that at the current threshold value, children are at risk from the arsenic content of rice and rice products, even assuming the mean daily intake of inorganic arsenic (5.4 g); this risk is apparent in younger children (9.25 kg bw) even at the lowest assumed iAs share (67.7%), and at 72.7% in older children (15 kg bw) (Figure 2). No such risk was found for adults.

Since the value of each of the risk assessment parameters depends on EDI, significant differences in their values were found between age groups. Negative correlations were observed between consumer body weight and EDI, THQ, and LCR, and positive correlations were observed between body weight and MoE.

The remaining EDI, THQ, MoE, and LCR scores corresponding to scenarios 2–4 are provided in Appendix A (Table A2, Table A3 and Table A4).

**Figure 3:**
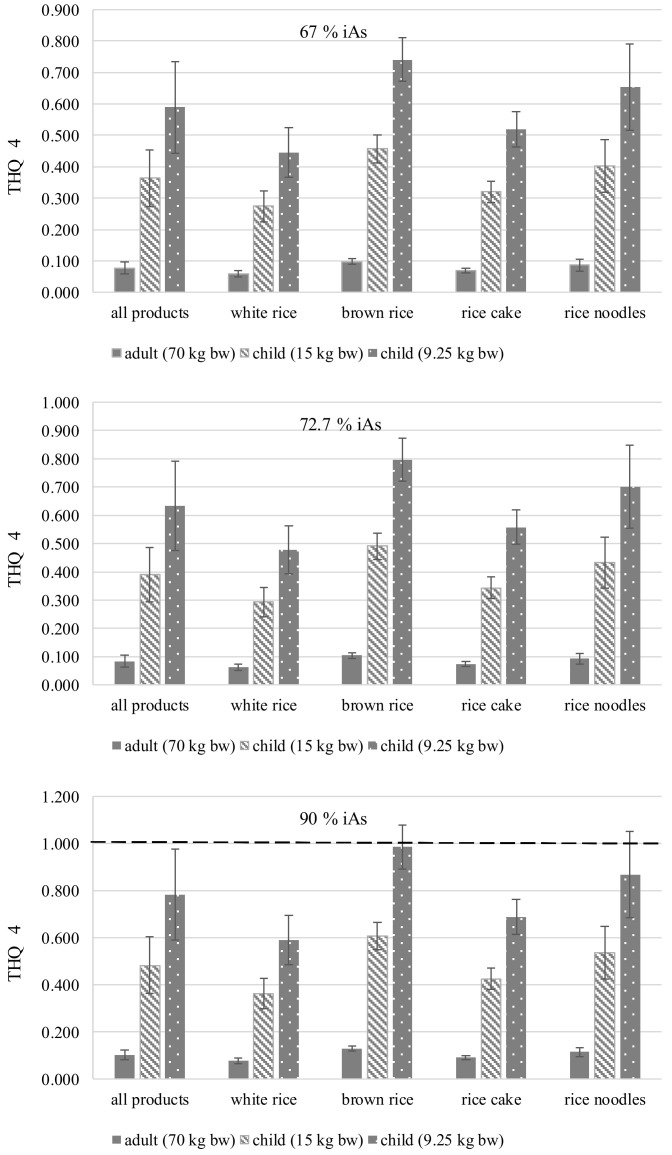
Total Hazard Quotient (THQ 4) based on iAs in rice and rice-based products accounting for 67.7%, 72.7%, and 90% of tAs (Mean ±SD). THQ 4 corresponds to scenario 4: threefold higher consumption than the mean, i.e., 16.4 g d^−1^ and maximum As contents in rice and rice-based products. The dashed line indicates the threshold. Systemic effects may occur at THQ > 1, i.e., the value is higher than the reference dose (RfD).

The results of the Total Hazard Quotient for scenario 1 (THQ 1), which are obtained for all tested products in general, ranged from 0.078 to 0.784, depending on the consumer group (Table 6). Depending on the adopted scenario, THQ results ranged from 0.047 (minimum THQ 1 for adults) to 1.197 (maximum THQ 4 for the youngest children). Figure 3 shows the detailed results calculated according to scenario 4 (THQ 4). In this example, the safety threshold, i.e., THQ = 1 was exceeded in the case of rice noodles and brown rice by small children (9.25 kg bw). When THQ > 1, i.e., THQ is higher than the reference dose (RfD), i.e., 0.3 µg kg^−1^ bw d^−1^, systemic effects may occur [31].

**Figure 4:**
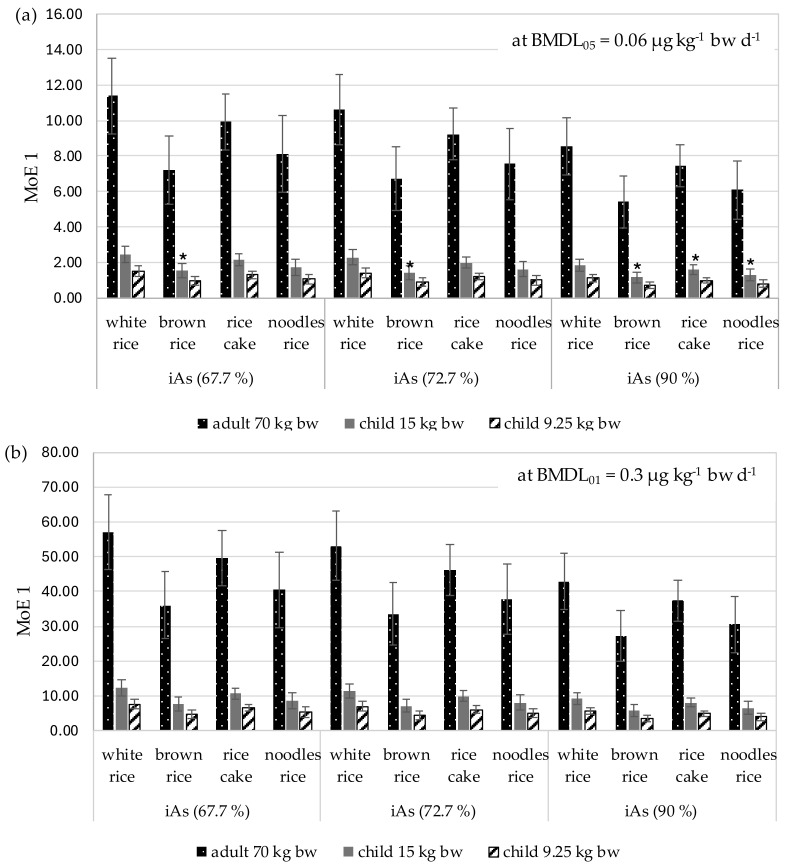
Margin of Exposure (MoE 1) calculated with respect to different BMDL values for iAs (1st scenario; Mean ± SD). * MoE < 1; MoE calculations were made on the basis of the following: (**a**) The currently-applicable BMDL_05_ for iAs [13]; (**b**) The previously-applicable BMDL_01_ [11]. MoE 1—MoE calculated for scenario 1 (mean consumption of rice products in Poland [8] and iAs being 67.7%; 72.7% and 90% of tAs).

Figure 4 shows the margin of exposure (MoE) results; these were calculated based on the mean consumption of rice and rice products in Poland (5.4 g d^−1^) and iAs values obtained from the mean total arsenic content in rice and rice products (scenario 1, Table 4). The remaining MoE results, i.e., MoE 2–4, are presented in Appendix A (Table A2–A4). The use of the BMDL_05_ value currently recommended by EFSA [13] resulted in significantly lower MoE values compared to calculations based on the previously-applicable BMDL_01_ value [11] (Figure 4). For example, the consumption of brown rice by a small child (9.25 kg) resulted in MoE values below 1, even at the lowest iAs values (67.7% of tAs). In the same group of consumers, MoE < 1 was also obtained for the consumption of rice noodles with an iAs content of 135.9 µg kg^−1^, i.e., assuming 90% of tAs (Table 5). In the MoE calculations based on the previously-applicable BMDL_01_ value (Figure 4b), all values were above 1. The MoE score ranged from 2.51 (rice noodles, iAs 90%, child weighing 9.25 kg) to 82.72 (white rice, iAs 67.7%, adult weighing 70 kg).

Lifetime Cancer Risk values (LCR 1) varied depending on the consumer group and iAs. The mean values ranged from 1.1 × 10^−5^ (adults, iAs 66.7%) to 1.1 × 10^−4^ (children weighing 9.25 kg, iAs 90%; Table 6). The remaining LCR results are provided in the Appendix A (Table A2, Table A3 and Table A4).

## 4. Discussion

### 4.1. Total Arsenic Content (tAs) in Rice and Rice Products

Most plants accumulate arsenic, but rice accumulates up to 10 times more than any other cereal crop under the same soil conditions [24,35]. This is due to the anaerobic growth environment of rice and its unique physiology [35]. Anaerobic conditions result in higher arsenic mobilization rates and greater bioavailability to rice [36]. An EFSA study [11] found the mean levels of total arsenic in rice grains and rice-based products available on the EU market to range from 140 to 170 µg kg^−1^. In this study, the range of mean tAs contents in rice and rice products was slightly wider, i.e., 104.3 µg kg^−1^ in white rice and 168.1 µg kg^−1^ in brown rice, with their maximum values being comparable (Table 5). However, other authors report higher results, e.g., Rahman et al. [6] found the mean arsenic content in white and brown rice to be 3.4 to 6.6 times higher. In the present study, the tAs content of white and brown rice was found to range from 69.5 to 227.8 µg kg^−1^, which is a much narrower range than noted in previous studies, i.e., from 58 to 406 µg kg^−1^ [37]. The mean tAs content in white rice (104.3 µg kg^−1^) was found to be 1.6 times lower (*p* < 0.05) than in brown rice (168.1 µg kg^−1^) (Table 5).

A comparable ratio of 1.5 between tAs levels in brown rice and white rice was reported by the FDA Center for Food Safety and Applied Nutrition [30]. Naito et al. [38] found 141 µg kg^−1^ of tAs and 131 µg kg^−1^ of iAs (93%) in white rice and 226 µg kg^−1^ and 208 µg kg^−1^ of tAs and iAs (92%) in brown rice from Japan. Various other studies have also noted a higher mean content of tAs in brown than in white rice grains [9,30,32,37,39,40], with Guillod-Magnin et al. [9] reporting 182 and 136 µg kg^−1^. The difference in As content between white and brown rice has been attributed to the removal of the outer hard bran layer of the grain in white rice production, which takes away some of the arsenic [17,35]. Indeed, Naito et al. [38] report that the level of total and inorganic arsenic in white rice samples depended on the degree of polishing (%); in brown rice, removal of 10% of the mass in the form of bran lowered tAs levels by 61–66% and iAs by 51–70%. However, Torres-Escribano et al. [37] report no significant difference between the mean tAs content in white rice (181 µg kg^−1^) and brown rice (199 µg kg^−1^).

Although brown rice generally accumulates more arsenic than white rice, As contamination is also reported in white rice. The degree of contamination in rice grain depends on the geographical location of the rice-growing area and the level of arsenic contamination of water [35]. Considerable variation in rice grain arsenic level has been noted in the US, where the South-Central region has almost double the mean arsenic concentrations (300 µg kg^−1^) of California (170 µg kg ^−1^) [41]. Our present findings also confirm that rice originating from the same country can differ in As content. Samples RLe and RKo from Myanmar differed significantly in tAs (*p* = 0.0013), as did RK and RA from Italy (*p* = 0.0012; Figure 1). However, brown rice product samples RbSa and RbDF from Pakistan demonstrated comparable arsenic content (Figure 1). Previous researchers report up to seven-fold variation in arsenic levels in rice depending on its origin, with the lowest content recorded in rice from Egypt (0.04 mg kg^−1^) and India (0.07 mg kg^−1^), and the highest from the USA (0.25 mg kg^−1^) and France (0.28 mg kg^−1^) [42]. 

When we compared rice products, significantly higher mean As contents (*p* < 0.05) were noted in rice noodles (151.0 µg kg^−1^) compared to white rice (104.3 µg kg^−1^), which is the general raw material for the production of rice flour. The tAs value in rice cakes was comparable to that of white rice but significantly lower than in brown rice (*p* = 0.0935). In contrast, the EFSA reports a higher mean arsenic content in rice cakes (99 µg kg^−1^) than in white rice (88 µg kg^−1^) [16]. Other studies also indicate that arsenic concentration is dependent on the rice percentage in the final product [9,15].

The majority of arsenic in rice products is inorganic As (75.2–90.1%) [29]. Williams et al. [43] found the share of iAs to be 64%, 80%, and 81% in European, Bangladeshi, and Indian rice, respectively. In most cases, the iAs level is assumed to be around 80% of total As or estimated from regression equations [31]. This is confirmed by the WHO report [24] for South Asia, where the iAs content in rice is typically about 80% tAs, with a median concentration of 0.1 mg kg^−1^, although in highly affected areas, this may exceed 0.2 mg kg^−1^. Guillod-Magnin et al. [9] found about 60–80% of the total arsenic content in rice and rice-based products to be As(III). The EFSA [44] reports the mean level of iAs in rice and rice products to be 71% (68–73%) of reported tAs levels. This value is almost fully consistent with those obtained for 42 types of rice by Menon et al. [32]. In some areas endemic for As, rice grains can contain over 90% inorganic As [20,30,34,45]. As such, the present study estimated the level of inorganic arsenic in the tested products based on three different percentage shares of iAs: 67.7%, 72.7%, and 90%. The calculated iAs levels ranged from 47.0 µg kg^−1^ (white rice, assuming 67.7% iAs) to 205.0 µg kg^−1^ (rice noodles, assuming 90% iAs) (Table 5). The FDA Report [30] found the maximum iAs values in different types of white rice grains to range from 20 to 249 µg kg^−1^; these values were almost 50% higher than those obtained in the present study. In contrast, the maximum values in brown rice were 32 to 249 µg kg^−1^. i.e., only 20% higher [30].

The BfR [15] found that for children, the highest intake of inorganic arsenic through the consumption of rice products came from the consumption of rice cakes and instant rice-based baby food. In the present study, the mean tAs level in rice cakes was 118.3 µg kg^−1^ (79.0–150.3 µg kg^−1^), with iAs ranging from 53.6 to 135.3 µg kg^−1^ depending on the assumed percentage share (Table 5). Islam et al. [46] report tAs levels ranging from 58.1 to 384.4 mg kg^−1^ in rice cakes made from 100% whole grain brown rice grown in Australia. Other authors [9] reported higher tAs mean values of 154 and 267 µg kg^−1^ in rice crackers and rice cereals, which are also almost exclusively rice-based. Signes-Pastor et al. [12] found iAs content in rice crackers to range from 19 to 212 µg kg^−1^ and showed a good correlation with the concentration in rice, indicating that most of the iAs originated from rice. In addition, the mean tAs was found to differ between puffed rice imported from New Zealand (213.3 µg kg^−1^) and from Australia (76.6 µg kg^−1^) [46].

### 4.2. Risk Assessment

The assessment of arsenic exposure via water is relatively simple: consumption of 1.5 L of drinking water per day containing 100 ppb As will exceed 130 µg day^−1^. However, exposure via food varies greatly depending on dietary habits: in heavily-polluted rice-growing areas in South Asia, the cumulative exposure can exceed 1000 µg d^−1^ [40]. In the European Union countries, high levels of arsenic in drinking water are rare [47]. For populations without elevated levels of arsenic in drinking water, rice is the largest source of arsenic in the diet [11,42]. Of American children between one and six years old who have the highest dietary exposure to arsenic (i.e., 95th percentile), rice and rice products contribute about 50% of their exposure [48].

The uptake of arsenic from rice and rice products depends on many factors, including its concentration, rice variety, consumption behavior, and cooking practices [49,50]. For example, white British people were found to consume a higher amount of rice per portion, but less frequently, than the rest of the ethnic group [51]. According to the FDA [30], the risk of arsenic exposure and associated health condition(s) increases proportionally with consumption and depends on the type of rice consumed. The mean consumption rate of rice varies greatly between countries, i.e., from 0.9 to 650 g per person per day [21]. The mean daily consumption of raw rice in Poland is only 5.4 g d^−1^ [8], while this value is three times higher in Spain (16 g person^−1^ day^−1^) and even 125 times higher in Asian countries (650 g person^−1^ day^−1^) [21,37]. The present study considered four scenarios to assess the risk to human health from arsenic intake from rice and rice products (Table 4). The first two scenarios are based on the mean rice consumption per person in Poland (i.e., 5.4 g d^−1^) [8] and the average iAs concentration in the tested products: these values are 91.7 µg kg^−1^; 98.5 µg kg^−1^ and 121.9 µg kg^−1^, assuming iAs shares of 67.7%; 72.7% and 90%. In the second scenario, iAs was calculated according to the maximum tAs content found in the tested products; thus, the following maximum iAs concentrations were obtained depending on the assumed share of iAs: 154.2 µg kg^−1^; 165.6 µg kg^−1^ and 205.0 µg kg^−1^ (Table 5).

Calculated Estimated Daily Intake (EDI 1) corresponded to the mean intake of rice products in Poland and the mean iAs content, ranging from 0.008 for adults to 0.071 µg kg^−1^ bw d^−1^ for children < one year of age (Table 6). The highest value was 0.235 µg kg^−1^ d^−1^, observed for infants in scenario 4 (EDI 4) (Table A4, Appendix A). Other authors [47] obtained higher results of the maximum daily intake of iAs in the case of infants consuming gluten-free infant rice (0.41 µg kg^−1^ d^−1^). Moreover, higher results of daily intake of inorganic arsenic, ranging from 0.38 to 1.92 µg kg^−1^ bw, were also shown by Islam et al. [52]. A WHO [24] report found the cumulative average daily intake (ADI) of arsenic in South and Southeast Asia to be more than twice the previous FAO/WHO guideline of 130 µg d^−1^ without exceeding the maximum recommended limits for arsenic in water or food. This value for an adult weighing 70 kg would correspond to an EDI of 1.86 µg kg^−1^, which is also significantly higher than that obtained in the present study. Rahman et al. [6] found the consumption of rice with As content ranging from 570 to 690 µg kg^−1^ to deliver from 250 to 360 µg As to the consumer per day. Assuming the body weights of the groups used in the present study (viz. 70 kg, 15 kg, and 9.25 kg), this would correspond to much higher EDI values (µg kg^−1^ d^−1^) amounting to 3.6–5.1 for adults, 16.7–24 for older children and 27–38.9 for younger children. Compared to the cited studies, our results seem to be very low. Nevertheless, even in scenario 1, and with the lowest assumed share of iAs (67.7%), the intake of iAs from brown rice by children from the infant group (9.25 kg bw) exceeds the permitted threshold based on the BMDL_05_ value (0.06 µg kg^−1^ bw d^−1^) currently recommended by EFSA [13] (Table 7). In older children (15 kg bw), the value was exceeded in scenario 3, assuming a 72.7% share of iAs (Table A3, Appendix A).

However, based on the previously-applicable BMDL_01_ value (0.3 µg kg^−1^ bw d^−1^) [44], even for the highest share of iAs (90%), the calculated EDI 1 values would constitute 5.2% of the BMDL_01_ for adults, 24.6% for older children and 39.9% for younger children, thus indicating a lack of risk in scenario 1. Furthermore, the previously applicable BMDL_01_ value was not exceeded for any of the consumer groups or scenarios considered. For adults, consumption of rice and rice products did not result in iAs intake being exceeded also for the current BMDL_05_ value (Table 6 and Table 7; Figure 2; Table A2, Table A3 and Table A4, Appendix A).

According to BfR [15], assuming mean consumption levels (4 g day^−1^, corresponding to approximately half a rice cake per day or 0.424 g kg^−1^ bw day^−1^, basis: consumers), children aged between six months and one year age demonstrate the highest exposure to inorganic arsenic in proportion to their body weight, i.e., 0.11 μg kg^−1^ bw day^−1^, corresponding to MoE values of 3 to 73 calculated for BMDL_01_ values from 0.34 to 0.69 μg kg^−1^ bw d^−1^ [10]. These values are comparable to those obtained in this work (Figure 3).

The comparison of obtained EDI results indicates that at the current threshold value (BMDL_05,_ [13]), the daily intake of inorganic arsenic poses a risk to children: younger children (9.25 kg bw; < 1 year old) at an iAs level of 67.7% and older children (15 kg bw; under three years old), at an iAs of 72.7% (Figure 2). However, even assuming an iAs share of 90%, significantly lower EDI 1 values were obtained (5.2%, 24.6%, and 39.9% for adults, older and younger children, respectively) when matched against the previously-acceptable threshold (BMDL_01_).

The new BMDL_05_ value [13] is more restrictive and returns considerably lower MoE values. As these results are not really comparable with data in previous studies based on the old value [31,32], the MoE was calculated based on both the new and the previously applicable BMDL_01_ values (Figure 3). In adults, the MoEs estimated in this way are low, ranging between 2 and 0.4 for typical consumers and between 0.9 and 0.2 for those consuming rice at the 95th percentile. MoE < 1 indicates an exposure level associated with a 5% increase in the incidence of skin cancer and, as such, may raise a health concern [13].

The margins between the estimated dietary exposure to iAs and the identified toxicological reference points are small for all age groups; as such, there is a strong need for risk management measures. For the younger age groups, the estimated exposure at high-level dietary iAs (95th percentile) is close to, or slightly exceeds, the lower BMDL_01_ of 0.3 μg kg^−1^ bw per day (MoE 1–2 for infants and young children, children, and adolescents) [14]. In a similar study conducted in Finland, Rintala et al. [53] found MoE ≤ 1 in both adult men and women, as well as in children consuming different rice products; however, this assumed the worst-case scenario, i.e., with the maximum iAs content in long-grain rice (0.28 mg kg^−1^) and children’s products (0.21 mg kg^−1^), and based on the lowest BMDL_01_ (0.3 µg kg^−1^ bw d^−1^).

Our MoE results calculated for the old BMDL_01_ values are higher than the estimates given by Hackethal et al. [14]. However, it should be noted that some of the MoEs obtained in this study for the youngest children are around or below 1 (Figure 3); as such, such products may pose a health risk based on the new BMDL_05_ values [13]. The new Reference Point (0.06 µg iAs kg^−1^ bw d^−1^) identified by the CONTAM Panel for skin cancer is within the range of mean dietary exposure estimates for iAs (0.03–0.15 µg kg^−1^ bw d^−1^) and below any of the 95th percentile exposure estimates (0.07–0.33 µg kg^−1^ bw d^−1^) for adults in Europe. Therefore, in this group, the MoEs range between 2 and 0.4 for mean consumers and between 0.9 and 0.2 at the 95th percentile exposure. The mean value obtained for adults in the present study (MoE 1 = 2.06) corresponds to the highest value reported by EFSA. An MoE of 1 describes the exposure level that could be associated with a 5% increase relative to the background incidence of skin cancer based on the available data. Despite the uncertainties, the CONTAM Panel concludes that these MoEs raise a health concern [13]. 

Regarding the intake of inorganic arsenic from rice and rice products by younger children (9.25 kg bw), the mean MoE 1 value calculated based on 90% iAs was 0.91 (Table 6). Consistently, the MoE fell with increased consumption and higher iAs values, reaching a mean value 3.7 times lower than 1 in scenario 4 (MoE 4 = 0.27 for infants). In all scenarios, the MoE values for adults were above the threshold (average 9.16–2.06; range 16.54–1.26).

Previously, MoE results were calculated based on the BMDL_01_ value, which was up to 5-fold higher than the new version (0.3 μg kg^−1^ bw d^−1^) [11], which would prevent any comparison with our present findings. Therefore, another set of MoE values was calculated based on BMDL_01_ (Figure 4). Based on these data, in scenario 1, the mean MoE ranged from 45.55 for adults to 4.55 for children less than one year of age (9.25 kg bw) (Figure 4). Moreover, even the mean values obtained in scenario 4 were above 1, ranging from 2.7 to 1.36. These values are still lower than those identified by the German Federal Institute for Risk Assessment (BfR), which reported MoE values of 9 to 500 for adults and 37 to 1000 for children at medium intake, and 2 to 143 for adults and 12 to 320 for children at high intake levels [P95] [15]. The BfR considered obtained MoE values to be relatively low. 

According to Liao et al. [31], only one-third of studies on carcinogenic risk assessment are based on measured concentrations of iAs. In the present study, Life Time Risk (LCR) was found to vary depending on the consumer group and the percentage level of calculated iAs in the tested products (Table 6; Table A2, Table A3 and Table A4, Appendix A). In scenario 1, in the present study, the lowest LCR (LCR1), 1.1 × 10^−5^, was obtained for adults (70 kg bw), assuming a 67.2% share of iAs, and the highest, 1.1 × 10^−4^, was calculated for infants (9.25 kg assuming a 90% share of iAs (Table 6).

For the worst-case scenario, i.e., scenario 4, which assumed a three-fold higher consumption of rice and rice products, and the highest mean iAs content (90% tAs), the mean LCR value (LCR 4) calculated for infants was 3.53 × 10^−4^ and the maximum 5.39 × 10^−4^. These results exceed the upper limit of 1 in 10,000 set by the US Environmental Protection Agency (EPA) by 3.53 to 5.39 times, the upper limit being 100 times higher than the commonly-accepted cancer risk associated with environmental carcinogens, i.e., one in a million persons [54].

The limit for LCR was also exceeded in scenarios 3 and 4 in children below three years old (15 kg bm) but was safe for adults (Table 6; Table A2, Table A3 and Table A4, Appendix A). These analyses assumed that children under five years of age consume up to three times more rice per kilogram of body weight compared to adults (16.2 g kg^−1^) [10,11,46]. Therefore, this sub-population is much more vulnerable to arsenic exposure than adults [46]. Children and adolescents are at greater risk of arsenic toxicities than adults due to their physiological and nutrition requirements, such as their high energy intake and fluid allowances per body weight [55]. Furthermore, they are a very important target for the food industry when selling rice products [56]. Rice is increasingly used as an ingredient in baby foods because of its soft taste, hypoallergenic properties, and easily digested carbohydrates. This is why there are large differences between adults and toddlers in risk assessments [57]. According to Liao et al. [31], although humans may not be consuming rice during infancy, many infant foods are rice-based and contain As. They obtained higher mean LCR results of 5.28 × 10^−4^ (max. 7.51 × 10^−4^) for both male and female consumers, these results being five times above the upper limit of 1.0 × 10^−4^. The authors note that due to differences in body weight and ADC, the calculated maximum LCR for female consumers was even higher, i.e., 8.02 × 10^−4^, which is eight times above the upper limit of the LCR. Other studies have found even higher LCR values [52], high energy, and fluid allowances, with the incremental lifetime cancer risk (ILCR) for rice consumers ranging from 0.57 × 10^−3^ to 2.88 × 10 ^3^ depending on region, within the Middle East, and from 0.54 × 10^−3^ to 2.12 × 10^−3^ depending on the variety. The group most at risk of cancer was found to be children (2–10 years). Furthermore, the authors found that women were more susceptible than men [52].

To avoid a significant risk of developing cancer (LCR > 1), a child weighing 9.25 kg cannot ingest more than 0.555 µg iAs d^−1^, which results from the BMDL_05_ value of 0.06 µg kg^−1^ bw d^−1^. Assuming mean consumption of rice and rice-based products (5.4 g d^−1^) and mean iAs content (91.7–121 µg kg^−1^) (Table 5), a child weighing 9.12 kg bw should avoid consuming more than 4.6–6.1 g of rice products per day, i.e., 32–46.9 g per week. Menon et al. [32] calculated that infants up to one year of age must be restricted to a maximum of 20 g of 28 rice types per day to avoid a risk of cancer, based on the MoE.

Recognizing the risk of arsenic exposure through food, some countries and agencies (e.g., China, WHO, and the European Union) have set limits for arsenic in food [24]. The maximum permissible levels of inorganic arsenic, according to EU Commission Regulation [18], are 0.15 mg kg^−1^ for polished or white rice, 0.25 mg kg^−1^ for parboiled rice, and 0.1 mg kg^−1^ for rice intended for the production of food for infants and young children. For rice products, the maximum residue limits (MRLs) are 0.3 mg kg^−1^ for rice cake and 0.25 mg kg^−1^ for rice flour. At assumed iAs levels of 67.7% and 72.7%, our findings (Table 5) indicate that even the maximum As content was lower than the EU-recommended levels [18].

Considering the maximum allowable levels specified for products intended for children, only white rice (iAs 67.7% of tAs) was found to be safe for consumption, but even at the maximum determined As level. Malabadi et al. [35] found that 28 of 55 tested types of rice exceeded the maximum limit for food intended for infants [18] and were not suitable for the production of food products for children or for direct feeding. Our calculations indicate that the iAs level in white rice is below the maximum allowable limit for infants (100 µg kg^−1^) for all three assumed shares; in the case of the maximum values, this was only the case for the 67% share of iAs (98.3 µg kg^−1^).

The FDA [30] reported that of 42 tested rice varieties, 14 iAs levels were below the MRL for infants, with a mean content of 82 µg kg^−1^; this level increased to 152 µg kg^−1^ in the remaining 28 samples. Given the large global variations in consumption patterns, focusing on standards not based on local dietary practices can be a serious mistake [24]. Kollander et al. [58] report that out of a number of studied rice products, only one rice cake intended for consumption by infants and young children did not exceed the iAs MRL value for these groups. Since 2015, the Swedish Food Agency has advised that rice cakes should not be given to children under the age of six [59].

## 5. Conclusions

The overall rice intake by Polish consumers is very low, and even where it is three times higher (as is assumed in scenarios 3 and 4), it is significantly different from the mean intake in Asian countries. Therefore, even when assuming that the arsenic levels in rice and rice-based products are comparable to those given in previous studies, the EDI results obtained herein are many times lower. As such, the risk of taking in excessive amounts of arsenic is small or non-existent, especially in adult Polish consumers. However, the risk assessment in this study, based on new EFSA guidelines, including BMDL_05_, showed that—assuming maximum obtained arsenic levels in rice and rice-based products and an intake three times higher than average—Polish children below one year of age are exposed to high iAs intake, which may represent a health risk. Therefore, especially for infants and young children, further studies based on total diet and in line with national recommendations are needed. It would also be important to determine whether factors related to rice consumption behavior, cooking practices, knowledge about arsenic, and perceptions related to arsenic exposure in rice influence its intake.

## Figures and Tables

**Table 1:** Characteristics of the tested rice and rice products.

Product	Symbol *	Origin **	Description of Grain	Product	Symbol *	Origin **	Description
white rice n = 30	RSa	Pakistan	long	rice cakes n = 30	CK	Italy	natural; brown rice
	RK	Italy	medium		CGf	ns	natural; brown rice
	RLe	Myanmar	long; parboiled		CSo	ns	natural; brown rice
	RKP	ns	long grain		CLe	ns	natural; brown rice
	RKo	Myanmar	medium		CVf	ns	natural; brown rice
	RA	Italy	long; parboiled		CSa	ns	natural; white and brown rice
brown rice n = 30	RbSa	Pakistan	long	rice noodles n = 30	NSu	Thailand	rice flour 90%, water 10%
	RbLi	Paraguay	long		NTv	Thailand	rice noodles
	RbK	Italy	short; natural whole		NFa	Thailand	ribbon 5 mm; rice flour, water
	RbLe	Gujana	long		NDc	Vietnam	natural
	RbKP	ns	medium; whole; nutty flavor		NMd	Vietnam	natural
	RbDF	Pakistan	least processed		NDG	Thailand	vermicelli; rice flour 90%, water

* The symbols indicate the group of product—white rice (R), brown rice (Rb), rice cakes (C), and rice noodles (N)—and the remaining letters refer to the manufacturer; ** refers to the origin of rice; n—number of samples; ns—not specified on the label.

**Table 2:** Operating parameters of the MDS 2000 microwave furnace.

Parameter	Stage				
	1	2	3	4	5
Power (%) *	85	85	100	100	0
Pressure (PSI) **	20	40	85	150	0
Time (min)	10	10	10	10	0
Fan Speed	100	100	100	100	100

* Device power 630 W (100%); ** 1 PSI = 6894.75 Pa.

**Table 3:** HG-ICP–AES operating conditions.

Parameter	Value (Unit)
As analytical line	193.759 nm
Argon flow rate (plasma)	12 L min^−1^
Nebulizer flow rate	0.7 L min^−1^
Sample solution flow rate	1 mL min^−1^
NaBH_4_ flow rate	0.7 mL min^−1^
HCl flow rate	0.6 mL min^−1^

**Table 4:** Consumer exposure scenarios for iAs intake from rice and rice products.

Scenario	Consumption Rates * (g day^−1^)	tAs Content in Selected Rice Products		The Percentage Share of iAs
		Mean	Maximum	
1	5.4	x		67.7%; 72.7%; 90% of tAs
2	5.4		x	
3	16.2	x		
4	16.2		x	

* Scenario 1 and 2—the current per capita consumption rates of rice products in Poland [8]; Scenarios 3 and 4—consumption rates three times the mean value in Poland.

**Table 5:** Total and inorganic arsenic content in the tested rice and rice-based products.

Product (n)	tAs (µg kg^−1^)	iAs (µg kg^−1^) *, Mean ± SD (Min–Max)		
		67.7%	72.7%	90%
white rice (30)	104.3 ^a^ ± 19.6 (69.5–145.3)	70.6 ^a^ ± 13.3 (47.0–98.3)	75.8 ^a^ ± 14.2 (50.5–105.6)	93.9 ^a^ ± 17.6 (62.5–130.7)
brown rice (30)	168.1 ^b^ ± 34.8 (87.6–224.7)	113.8 ^b^ ± 23.5 (59.3–152.1)	122.2 ^b^ ± 25.3 (63.7–163.3)	151.3 ^b^ ± 31.3 (78.9–202.2)
rice cakes (30)	118.3 ^a^ ± 17.0 (79.0–150.3)	80.1 ^a^ ± 11.5 (53.6–101.8)	86.0 ^a^ ± 12.4 (57.6–109.3)	106.5 ^a^ ± 15.3 (71.3–135.3)
rice noodles (30)	151.0 ^c^ ± 37.6 (90.6–227.8)	102.3 ^c^ ± 25.5 (61.3–154.2)	109.8 ^c^ ± 27.4 (65.8–165.6)	135.9 ^c^ ± 33.8 (81.5–205.0)
all products (120)	135.4 ± 38.1 (69.5–227.8)	91.7 ± 25.8 (47.0–154.2)	98.5 ± 27.7 (50.5–165.6)	121.9 ± 34.3 (62.5–205.0)

n—number of samples; * The calculations assumed three levels of iAs in rice and rice products in relation to tAs: 67.7%, 72.7%, and 90%; ^a,b,c^ superscript letters indicate significant differences in As levels between product groups (Duncan test, *p* < 0.05).

**Table 6:** General parameters of iAs risk assessment based on scenario 1 *.

Parameter	Share of iAs in tAs	Mean ± SD		
		Adult, 70 kg bw	Child, 15 kg bw	Child, 9.25 kg bw
EDI 1 (µg kg^−1^ bw d^−1^)	67.7%	0.008 ^aA^ ± 0.002	0.033 ^aB^ ± 0.010	0.053 ^aC^ ± 0.015
	72.7%	0.008 ^aA^ ± 0.002	0.035 ^aB^ ± 0.010	0.057 ^aC^ ± 0.016
	90%	0.009 ^bA^ ± 0.003	0.044 ^bB^ ± 0.012	0.071 ^bC^ ± 0.020
THQ 1	67.7%	0.078 ^aA^ ± 0.019	0.364 ^aB^ ± 0.090	0.590 ^aC^ ± 0.146
	72.7%	0.084 ^aA^ ± 0.021	0.390 ^aB^ ± 0.097	0.633 ^aC^ ± 0.157
	90%	0.104 ^bA^ ± 0.026	0.483 ^bB^ ± 0.120	0.784 ^bC^ ± 0.195
MoE 1	67.7%	9.16 ^aA^ ± 2.53	1.96 ^aB^ ± 0.54	1.2 ^aC^ ± 0.33
	72.7%	8.53 ^aA^ ± 2.36	1.83 ^aB^ ± 0.51	1.13 ^aC^ ± 0.31
	90%	6.89 ^bA^ ± 1.90	1.48 ^bB^ ± 0.41	0.91 ^bC^ ± 0.25
LCR 1	67.7%	1.1 × 10^−5 aA^ ± 3 × 10^−6^	5.0 × 10^−5 aB^ ± 1.4 × 10^−5^	8.0 × 10^−5 aC^ ± 2.3 × 10^−5^
	72.7%	1.1 × 10^−5 aA^ ± 3 × 10^−6^	5.3 × 10^−5 aB^ ± 1.5× 10^−5^	8.6 × 10^−5 aC^ ± 2.4 × 10^−5^
	90%	1.4 × 10^−5 bA^ ± 4× 10^−6^	6.6 × 10^−5 bB^ ± 1.9× 10^−5^	1.1× 10^−4 bC^ ± 3.0 × 10^−5^

* Details of the assumptions for the first scenario are given in Table 4. Results were calculated for a mean daily intake of 5.4 g [8]. ^a,b^ Different lower case letters in superscript indicate a significant difference in the share of iAs in tAs; ^A,B,C^ different capital letters indicate significant differences depending on the of the consumer group (child 9.25 kg bw is a child between 6 months and <1 year-old; child 15 kg bw—child < 3 year-old).

**Table 7:** Estimated Daily Intake (EDI) of iAs from rice and rice products (1st scenario).

Share of iAs in tAs	Product	EDI (µg kg^−1^ bw d^−1^), Mean ± SD		
		Adult, 70 kg bw	Child, 15 kg bw	Child, 9.25 kg bw
67.7%	white rice	0.005 ^a^ ± 0.001	0.025 ^a^ ± 0.005	0.041 ^a^ ± 0.008
	brown rice	0.009 ^b^ ± 0.002	0.041 ^b^ ± 0.008	0.066 ^b^ ± 0.014
	rice cake	0.006 ^a^ ± 0.001	0.029 ^a^ ± 0.004	0.047 ^a^ ± 0.007
	rice noodles	0.008 ^c^ ± 0.002	0.037 ^c^ ± 0.009	0.060 ^c^ ± 0.015
72.7%	white rice	0.006 ^a^ ± 0.001	0.027 ^a^ ± 0.005	0.044 ^a^ ± 0.008
	brown rice	0.009 ^b^ ± 0.002	0.044 ^b^ ± 0.009	0.071 ^b^ ± 0.015
	rice cake	0.007 ^a^ ± 0.001	0.031 ^a^ ± 0.004	0.050 ^a^ ± 0.007
	rice noodles	0.008 ^c^ ± 0.002	0.040 ^c^ ± 0.010	0.064 ^c^ ± 0.016
90%	white rice	0.007 ^d^ ± 0.001	0.034 ^a^ ± 0.006	0.055 ^d^ ± 0.010
	brown rice	0.012 ^e^ ± 0.002	0.054 ^e^ ± 0.011	0.088 ^e^ ± 0.018
	rice cake	0.008 ^d^ ± 0.001	0.038 ^d^ ± 0.006	0.062 ^d^ ± 0.009
	rice noodles	0.010 ^f^ ± 0.003	0.044 ^f^ ± 0.012	0.079 ^f^ ± 0.020

The intakes were calculated based on the following assumptions: the mean consumption of rice and rice products in Poland was 5.4 g day^−1^ [8], and iAs made up 67.7%; 72.7%, and 90% of total arsenic, as detailed in scenario 1 (Table 4). ^a–f^ In the columns, the same lower case letters in superscript indicate no differences (*p* < 0.05) between the EDI calculated for individual products depending on the share of iAs in tAs (%).

## Data Availability

The original contributions presented in the study are included in the article, further inquiries can be directed to the corresponding author.

## Appendix A

**Table A1:** Total and inorganic arsenic content in rice and rice products according to literature dates.

Product	Mean			References
	tAs (µg kg^−1^)	iAs (µg kg^−1^)	Share of iAs in tAs (%)	
Italian rice varieties	210	96.2	48.5	[60]
different rice varieties	201.5	129.1	63.8	[47]
rice of different varieties	155	102	65.8	[61]
white rice	138.9	83.6	62.2	[62]
white rice	212.1	134	63.2	[63]
white rice	36	21	58.8	[64]
white rice (Australia)	242.3	171	94	[39]
white rice (Pakistan)	82.3	83	96	
white rice (Thailand)	172	168	92.5	
parboiled rice	64	62.5	97.7	
brown rice	33	23	69.6	[64]
brown rice (Australia)	307	227	62.5	[39]
brown rice (Canada)	28	19	68	
brown rice	107	97.3	57.6	[62]
brown rice	189.3	43.3	22.9	[65]
baby rice	268.2	172.6	64.4	[46]
baby rice	180.2	119.6	68.7	[12]
baby foods—dry form	83.2	67.5	81.1	[9]
infant rice (Spain)	126	69	69	[47]
rice cakes	204.3	205	78.7	[46]
rice wafers, conventional	130	106	81.5	[64]
rice cakes	235.9	120	78.7	[58]
noodles	190	120	86.3	[29]
rice noodles	92.6	79.78	75.5	[58]
rice crackers	132	108.3	82	[12]
rice crackers	380	205	75.4	[29]
rice crackers	168	134	79.8	[9]
rice crackers	239	181.7	40.2	[46]
rice cereals	149	112.7	75.6	[12]
rice cereals	278	204	73.4	[9]
crisped rice	210	120	86.4	[29]
puffed rice	270	140	76.7	[58]
puffed rice	76.6	64.8	97	[46]
**Mean of iAs in tAs (%)**			**72.7**	

**Table A2:** Summary assessment of the risk to human health resulting from the presence of arsenic in rice and rice products (2nd scenario).

Share of iAs in tAs	Parameter	Mean ± SD		
		Adult, 70 kg bw	Child, 15 kg bw	Child, 9.25 kg bw
67.7%	EDI 2 (µg kg^−1^ bw d^−1^)	0.008 ± 0.002	0.036 ± 0.009	0.059 ± 0.015
	THQ 2	0.026 ± 0.006	0.121 ± 0.030	0.197 ± 0.049
	MoE 2	8.20 ± 2.09	1.76 ± 0.45	1.08 ± 0.28
	LCR 2	1.17 × 10^−5^ ± 2.90 × 10^−6^	5.45 × 10^−5^ ± 1.35 × 10^−5^	8.84 × 10^−5^ ± 2.20 × 10^−5^
72.7%	EDI 2 (µg kg^−1^ bw d^−1^)	0.008 ± 0.002	0.039 ± 0.010	0.063 ± 0.016
	THQ 2	0.028 ± 0.007	0.130 ± 0.032	0.211 ± 0.052
	MoE 2	7.63 ± 1.95	1.64 ± 0.42	1.01 ± 0.26
	LCR 2	1.26 × 10^−5^ ± 3.12 × 10^−6^	5.86 × 10^−5^ ± 1.45 × 10^−5^	9.50 × 10^−5^ ± 2.36 × 10^−5^
90%	EDI 2 (µg kg^−1^ bw d^−1^)	0.010 ± 0.003	0.048 ± 0.012	0.078 ± 0.019
	THQ 2	0.035 ± 0.009	0.161 ± 0.040	0.261 ± 0.065
	MoE 2	6.17 ± 1.57	1.32 ± 0.34	0.81 ± 0.21
	LCR 2	1.55 × 10^−5^ ± 3.86 × 10^−6^	7.25 × 10^−5^ ± 1.80 × 10^−5^	1.18 × 10^−4^ ± 2.92 × 10^−5^

**Table A3:** Summary assessment of the risk to human health resulting from the presence of arsenic in rice and rice products (3rd scenario).

Share of iAs in tAs	Parameter	Mean ± SD		
		Adult, 70 kg bw	Child, 15 kg bw	Child, 9.25 kg bw
67.7%	EDI 3 (µg kg^−1^ bw d^−1^)	0.021 ± 0.006	0.099 ± 0.028	0.161 ± 0.045
	THQ 3	0.071 ± 0.020	0.330 ± 0.093	0.535 ± 0.150
	MoE 3	3.05 ± 0.84	0.65 ± 0.18	0.40 ± 0.11
	LCR 3	3.18 × 10^−5^ ± 8.95 × 10^−6^	1.49 × 10^−4^ ± 4.17 × 10^−5^	2.41 × 10^−4^ ± 6.77 × 10^−5^
72.7%	EDI 3 (µg kg^−1^ bw d^−1^)	0.023 ± 0.006	0.106 ± 0.030	0.172 ± 0.048
	THQ 3	0.076 ± 0.021	0.354 ± 0.100	0.575 ± 0.162
	MoE 3	2.84 ± 0.79	0.61 ± 0.17	0.38 ± 0.10
	LCR 3	3.42 × 10^−5^ ± 9.61 × 10^−6^	1.60 × 10^−4^ ± 4.48 × 10^−5^	2.59 × 10^−4^ ± 7.27 × 10^−5^
90%	EDI 3 (µg kg^−1^ bw d^−1^)	0.028 ± 0.008	0.132 ± 0.037	0.213 ± 0.060
	THQ 3	0.094 ± 0.026	0.439 ± 0.123	0.712 ± 0.200
	MoE 3	2.30 ± 0.63	0.49 ± 0.14	0.30 ± 0.08
	LCR 3	4.23 × 10^−5^ ± 1.19 × 10^−5^	1.97 × 10^−4^ ± 5.55 × 10^−5^	3.20 × 10^−4^ ± 9.00 × 10^−5^

**Table A4:** Summary assessment of the risk to human health resulting from the presence of arsenic in rice and rice products (4th scenario).

Share of iAs in tAs	Parameter	Mean ± SD		
		Adult, 70 kg bw	Child, 15 kg bw	Child, 9.25 kg bw
67.7%	EDI 4 (µg kg^−1^ bw d^−1^)	0.023 ± 0.006	0.109 ± 0.027	0.177 ± 0.044
	THQ 4	0.078 ± 0.019	0.364 ± 0.090	0.590 ± 0.146
	MoE 4	2.73 ± 0.70	0.59 ± 0.15	0.36 ± 0.09
	LCR 4	3.505 × 10^−5^ ± 8.702 × 10^−6^	1.636 × 10^−4^ ± 4.06 × 10^−5^	2.653 × 10^−4^ ± 6.585 × 10^−5^
72.7%	EDI 4 (µg kg^−1^ bw d^−1^)	0.025 ± 0.006	0.117 ± 0.029	0.190 ± 0.047
	THQ 4	0.084 ± 0.021	0.390 ± 0.097	0.633 ± 0.157
	MoE 4	2.54 ± 0.65	0.55 ± 0.14	0.34 ± 0.09
	LCR 4	3.76 × 10^−5^ ± 9.34 × 10^−6^	1.76 × 10^−4^ ± 4.36 × 10^−5^	2.85 × 10^−4^ ± 7.07 × 10^−5^
90%	EDI 4 (µg kg^−1^ bw d^−1^)	0.031 ± 0.008	0.145 ± 0.036	0.235 ± 0.058
	THQ 4	0.104 ± 0.026	0.483 ± 0.120	0.784 ± 0.195
	MoE 4	2.06 ± 0.52	0.44 ± 0.11	0.27 ± 0.07
	LCR 4	4.66 × 10^−5^ ± 1.16 × 10^−5^	2.17 × 10^−4^ ± 5.40 × 10^−5^	3.53 × 10^−4^ ± 8.75 × 10^−5^
